# Characterization of a pathway-specific activator of milbemycin biosynthesis and improved milbemycin production by its overexpression in *Streptomyces bingchenggensis*

**DOI:** 10.1186/s12934-016-0552-1

**Published:** 2016-09-07

**Authors:** Yanyan Zhang, Hairong He, Hui Liu, Haiyan Wang, Xiangjing Wang, Wensheng Xiang

**Affiliations:** 1 State Key Laboratory for Biology of Plant Diseases and Insect Pests, Institute of Plant Protection, Chinese Academy of Agricultural Sciences, No. 2 Yuanmingyuan West Road, Haidian District, Beijing, 100193 China; 2 School of Life Science, Northeast Agricultural University, No. 59 Mucai Street, Xiangfang District, Harbin, 150030 China

**Keywords:** Milbemycin, LAL, MilR, Overexpression, *Streptomyces bingchenggensis* BC04

## Abstract

**Background:**

Milbemycins, a group of 16-membered macrolides with potent anthelminthic and insecticidal activity, are produced by several *Streptomyces* and used widely in agricultural, medical and veterinary fields. Milbemycin A3 and A4, the main components produced by *Streptomyces bingchenggensis*, have been developed as an acaricide to control mites. The subsequent structural modification of milbemycin A3/A4 led to other commercial products, such as milbemycin oxime, lepimectin and latidectin. Despite its importance, little is known about the regulation of milbemycin biosynthesis, which has hampered efforts to enhance milbemycin production via engineering regulatory genes.

**Results:**

*milR*, a regulatory gene in the milbemycin (*mil*) biosynthetic gene cluster of *S. bingchenggensis*, encodes a large ATP-binding regulator of the LuxR family (LAL family), which contains an ATPase domain at its N-terminus and a LuxR-like DNA-binding domain at the C-terminus. Gene disruption and genetic complementation revealed that *milR* plays an important role in the biosynthesis of milbemycin. β-glucuronidase assays and transcriptional analysis showed that MilR activates the expression of the *milA4*-*E* operon and *milF* directly, and activates the other *mil* genes indirectly. Site-directed mutagenesis confirmed that the ATPase domain is indispensable for MilR’s function, and particularly mutation of the conserved amino acids K37A, D122A and D123A, led to the loss of MilR function for milbemycin biosynthesis. Overexpression of an extra copy of *milR* under the control of its native promoter significantly increased production of milbemycin A3/A4 in a high-producing industrial strain *S. bingchenggensis* BC04.

**Conclusions:**

A LAL regulator, MilR, was characterized in the *mil* gene cluster of *S. bingchenggensis* BC04. MilR could activate milbemycin biosynthesis through direct interaction with the promoter of the *milA4*-*E* operon and that of *milF*. Overexpression of *milR* increased milbemycin A3/A4 production by 38 % compared with the parental strain BC04, suggesting that genetic manipulation of this activator gene could enhance the yield of antibiotics.

**Electronic supplementary material:**

The online version of this article (doi:10.1186/s12934-016-0552-1) contains supplementary material, which is available to authorized users.

## Background

Milbemycins are a group of 16-membered macrolides that share a similar lactone ring with avermectin, the discovery of which won a Nobel Prize in 2015. Similar to avermectins, milbemycins have attracted considerable attention and have been developed as acaricides, insecticides and anthelmintics because of their excellent activities against a variety of nematodes, parasitic insects and arthropod parasites, and their low toxicities to the host organisms [1–4]. Milbemycins have been used to control mites, liriomyza, aphidoidea and aleyrodidae that have developed resistance to avermectin and organophosphorus pesticides on 24 plant species, including apple, citrus, strawberry and tea. Milbemycin A3/A4 show high aricidal activity and have been used widely to control mites since 1990 in developed countries [5]. Milbemycin oxime, a semi-synthetic analog of milbemycin A3/A4, has been used commercially against worms, insects and mites of pet animals in China, Europe, Japan and the USA [6, 7]. In addition, lepimectin, another milbemycin A3/A4 derivative, has also been developed and used widely in the agricultural field.

Milbemycins were first isolated from *Streptomyces hygroscopicus* subsp. *aureolacrimosus* in 1967 in Japan [8]. Thereafter, another milbemycin-producing strain, *Streptomyces bingchenggensis*, was isolated by our laboratory and used as an important industrial producer of milbemycins [9–11]. *Streptomyces bingchenggensis* produces milbemycin A3, A4, and four α-class and β-class C5-O-methylmilbemycins (B2, B3, β1 and β2) as the main components, together with the polyether nanchangmycin and trace amounts of new milbemycin analogs [9–12]. In 2010, the genome information of *S. bingchenggensis* was published (GenBank Accession No. CP002047) and the milbemycin biosynthetic gene cluster (*mil* gene cluster) was identified [13]. The *mil* gene cluster (SBI00726–SBI00790) contains 10 genes, which are homologs to those of avermectin gene cluster, including one putative regulatory gene (SBI_00734, *milR*), four multifunctional modular type I polyketide synthase components (PKS; *milA1*, *milA2*, *milA3* and *milA4*), four tailoring enzyme genes (*milC*, *milD*, *milE* and *milF*), a putative *orf1* with unknown function and a large (62 kb) insertion fragment between *milR* and *milA1*. However, the overall gene organization of the milbemycin gene cluster is different from that of avermectin, e.g. the *mil* cluster PKS genes (*milA1*, *milA2*, *milA3* and *milA4*) are organized into four operons and are transcribed independently, while the avermectin PKS genes are organized as two groups (*aveA1*–*aveA2* and *aveA3*–*aveA4*). Initially, the biosynthetic pathway of milbemycin was proposed based on physicochemical characterization, bioconversion data and some studies on the biosynthesis of avermectin and meilingmycin [14–16]. Seven malonyl-CoA units and five methylmalonyl-CoA units are condensed to the starting unit derived from acetate or propionate, catalyzed by the PKSs to form the polyketide backbone, in a stepwise process. The tailoring enzyme genes *milD* (encoding C5-O-methyltransferase) and *milF* (encoding C5-ketoreductase) from the cluster were also characterized. MilD is responsible for the methylation of the hydroxyl group on C-5 of milbemycin A3/A4 [17], while MilF catalyzes the reduction of the C-5 keto group in the biosynthesis of milbemycin [18]. However, no report has been published so far on the regulation of milbemycin biosynthesis. A better understanding of the regulation mechanisms will be useful to construct high-producing industrial strains [19, 20].

In this study, to dissect the underlying regulation mechanism of milbemycin biosynthesis in *S. bingchenggensis*, the only candidate regulatory gene in the *mil* gene cluster, *milR*, was characterized. The deduced product of this gene is a LAL family transcriptional regulator. *milR* is the homolog of *aveR*, which is the regulatory gene for avermectin biosynthesis. We demonstrated that MilR shows a different regulatory pattern compared with that of *aveR*. MilR controls the type I polyketide chain termination and modification steps of milbemycin biosynthesis by regulating *milA4*-*E* operon and *milF* promoters directly. Overexpression of *milR* by one extra copy significantly increased the production of milbemycin A3/A4.

## Results

### *milR* encodes a putative LAL transcriptional regulator

**Fig. 1:**
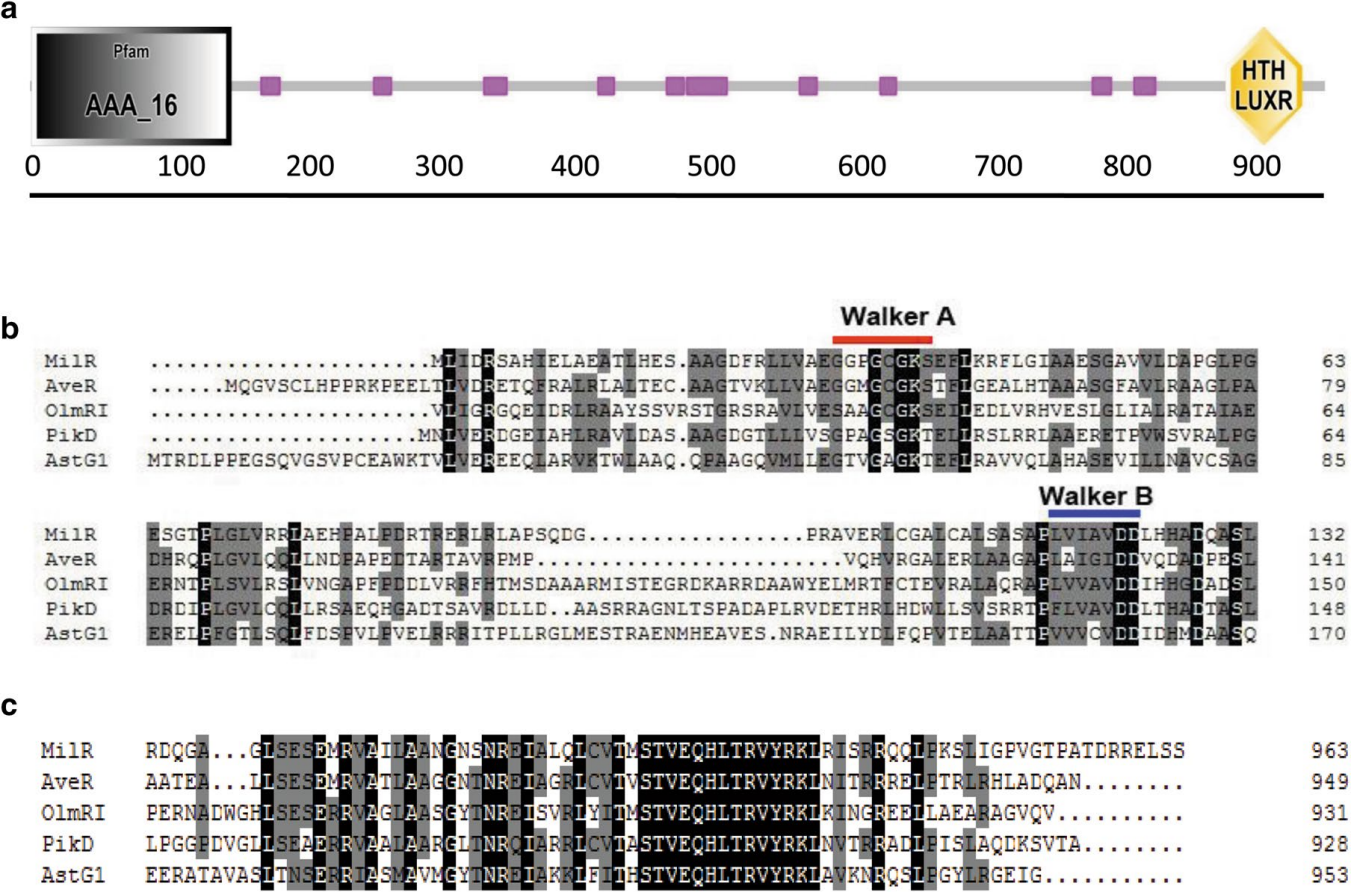
Domain structure and amino acid alignment of parts of the MilR. **a** Predicted domain structure of MilR. AAA_16: A domain abbreviation for ATPases associated with diverse cellular activities; HTH-LUXR: A DNA-binding, helix-turn-helix (HTH) domain of about 65 amino acids, present in transcription regulators of the LuxR family. **b** Alignment of the AAA domain of MilR with related proteins. *Numbers* indicate amino acid residues from the N-terminus of the protein. Identical amino acid residues are *black*, and similar residues are *shaded*. AveR, a cluster-situated regulator (CSR) of avermectin biosynthesis in *Streptomyces avermitilis*; OlmRI, a CSR of oligomycin biosynthesis in *Streptomyces avermitilis*; PikD, a CSR of pikromycin biosynthesis in *Streptomyces venezuelae*; AstG1, a CSR of ansatrienins biosynthesis in *Streptomyces* sp. XZQH13. The conserved Walker A and B motifs are indicated by *red* and *blue overlines* respectively. **c** Comparison of the HTH-LUXR domain of MilR with those of other proteins

*milR* encodes a putative polypeptide of 964 aa with a predicted molecular mass of 102.4 kDa. Bioinformatics analysis indicated that MilR is a LAL family (large ATP-binding regulator of the LuxR family) regulatory protein (Fig. 1a). The N-terminus of MilR contains an AAA (ATPases associated with diverse cellular activities) domain (amino acids 1–150) (Pfam No. PF00004), and the C-terminal portion contains an HTH-LuxR-like DNA-binding domain (amino acids 890–947) (SMART No. SM00421). MilR shows strong similarity to several regulatory proteins, such as AveR (49 % identity) and OlmRI (39 % identity) from *Streptomyces avermitilis*, PikD (37 % identity) from *Streptomyces venezuelae*, and AstG1 (35 % identity) from *Streptomyces *sp. XZQH13 [21–25].

The AAA domain of MilR belongs to the P-loop NTPases (nucleoside triphosphatases) superfamily, which is defined by the presence of a conserved nucleotide phosphate-binding motif, also known as the Walker A motif (GX_4_GK[S/T], where X is any amino acid), a glycine-rich sequence followed by a conserved lysine and a serine or threonine, and the second Walker B motif (ΨΨΨΨ[D/E], where Ψ is a hydrophobic residue) (Fig. 1b) [26, 27]. Proteins of this family exert their activity through assembly and disassembly that are driven by the ATP binding and hydrolysis cycle of the AAA domain [28].

Interestingly, one leucine in the C-terminal portion (amino acid 834) of MilR is encoded by the rare TTA codon. This indicated that the translation of *milR* is controlled by *bldA*, which encodes the tRNA responsible for translating TTA into leucine [29].

### *milR* is essential for milbemycin biosynthesis

**Fig. 2:**
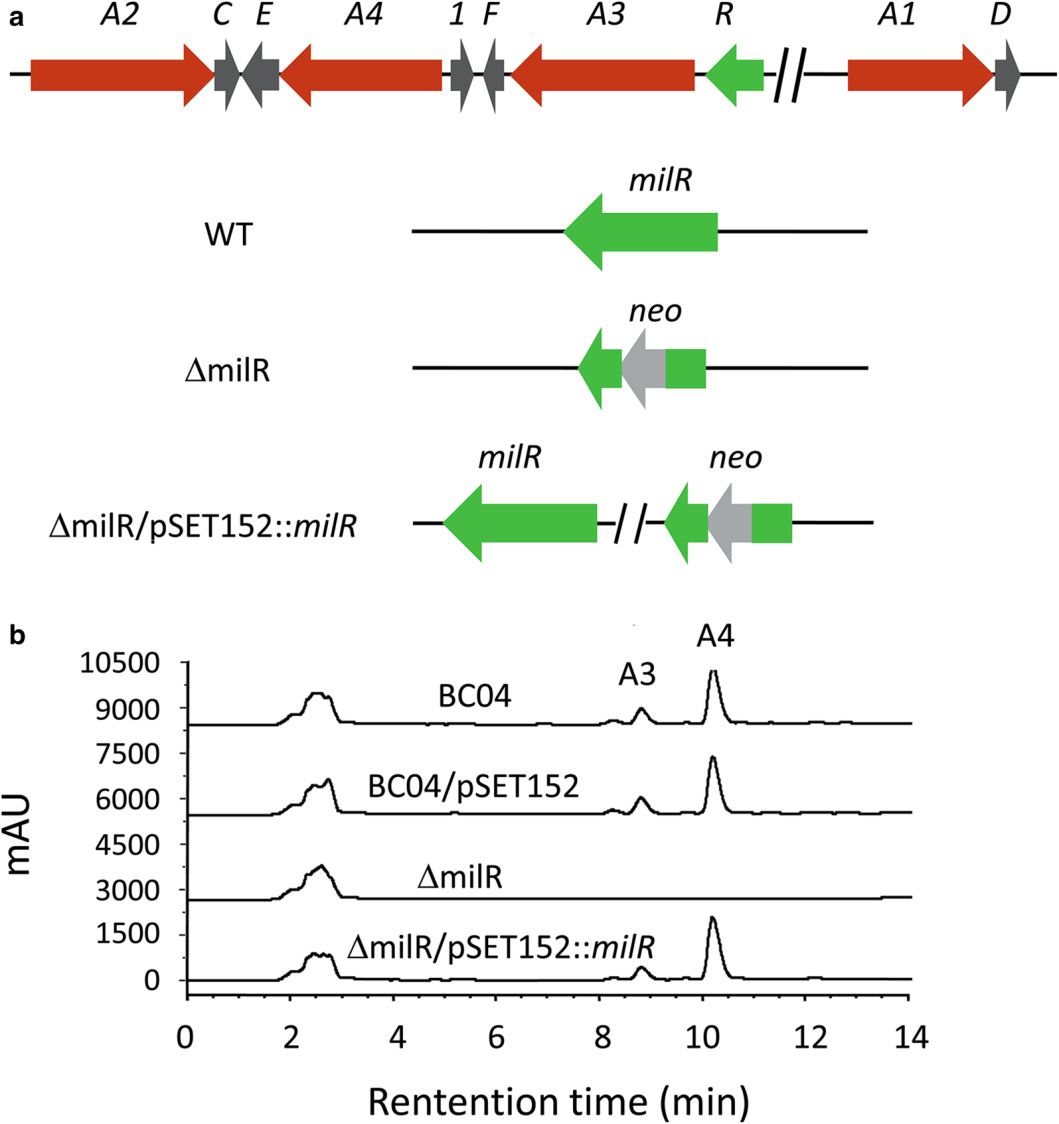
Effect of *milR* disruption on Milbemycin A3/A4 production. **a** Genetic organization of *mil* gene cluster in *Streptomyces bingchenggensis* BC04 and *diagram* of *milR* disruption and complementation constructions. *Each arrow* indicates a separate open reading frame (ORF) and orientation of transcription. **b** HPLC analysis of milbemycin A3/A4 production in *S. bingchenggensis* BC04, BC04/pSET152, ΔmilR, ΔmilR/pSET152::*milR*. Absorbance at 242 nm was monitored

To determine the role of *milR* in milbemycin biosynthesis, a *milR* disruption mutant (ΔmilR) was constructed via homologous recombination (Fig. 2a). In ΔmilR, a 1753-bp fragment internal to *milR* was replaced by the kanamycin resistance gene, *neo*. The resulting ΔmilR was further confirmed by PCR (Additional file 1: Figure S1). ΔmilR was cultured in fermentation medium for 9 days and the production of milbemycin was tested. The result showed that no milbemycin was produced by ΔmilR in comparison with BC04 and BC04/pSET152 controls (Fig. 2b). To verify that the phenotype was the result of *milR* disruption, a complementation experiment was carried out, in which an integrating plasmid, pSET152::*milR*, was used to complement ΔmilR. In pSET152::*milR*, *milR* was driven by its own promoter. Milbemycin production was restored in the complemented strain (ΔmilR/pSET152::*milR*) (Fig. 2b). These results demonstrated that MilR is indispensable for milbemycin production in *S. bingchenggensis*.

### MilR regulates the promoter regions of *milA4*-*E* operon, *orf1* and *milF* directly

**Fig. 3:**
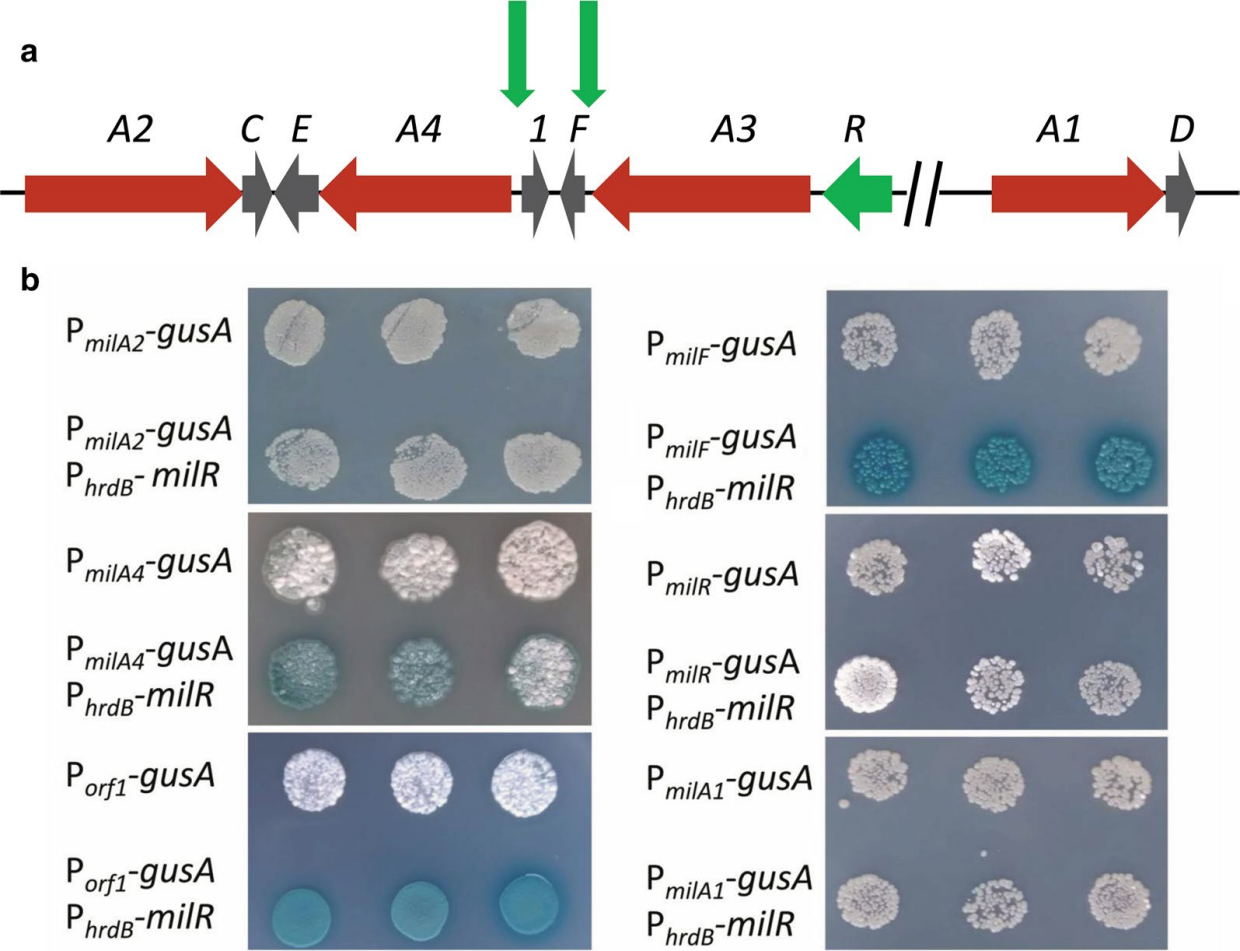
MilR directly activates promoters of *milA4*-*E*, *orf1* and *milF*. **a** MilR-regulating regions are indicated by two *vertical green arrows* in the *mil* gene cluster. **b** Chromogenic assays on AS-1 agar plates containing the substrate 5-bromo-4-chloro-3-indolyl-β-d-glucoronide. Data are representative of three independent experiments

*milR* is situated in the middle of the *mil* gene cluster; therefore, it is possible that the transcription of some *mil* genes might be regulated by MilR. To determine the potential targets of MilR, first, co-transcription analysis was performed to confirm the putative operons. Total RNAs were extracted from *S. bingchenggensis* BC04 after the onset of milbemycin production (3 days of cultivation in fermentation medium) and used as templates for cDNA synthesis and reverse transcription polymerase chain reaction (RT-PCR) analysis. Primers flanking four intergenic regions (regions 1–4) within the *mil* gene cluster were used: generation of PCR-amplified products by these primers would indicate transcription across the intergenic region (Additional file 2: Figure S2). The results showed that the *mil* gene cluster contains four transcriptional units (*milA2*-*C*, *milA4*-*E*, *milR*-*A3* and *milA1*-*D*) and two individually transcribed genes (*milF* and *orf1*). Based on the above results, the promoters of the six transcriptional units were then cloned separately upstream of *gusA* [encoding β-glucuronidase (GUS)]. The resulting plasmids were integrated into the ФC31 *attB* site of *Streptomyces coelicolor* M1146. At the same time, the coding region of *milR* was cloned downstream of P_*hrdB*_ in pIJ10500 to generate pIJ10500::P_*hrdB*_*milR*. pIJ10500::P_*hrdB*_*milR* was subsequently integrated into the ФBT1 *attB* site of the *S. coelicolor* M1146 derivatives containing the six different reporter constructs. Then *gusA* transcriptional fusions were assessed in agar-based chromogenic assays using 5-bromo-4-chloro-3-indolyl-β-d-glucuronide as the substrate (Fig. 3). None of the strains containing *mil* promoter::*gusA* plasmids that lacked constitutively expressed MilR gave GUS activity. When MilR was constitutively expressed, transcription of *gusA* from P_*milA4*_, P_*orf1*_ and P_*milF*_ was readily detected; however, transcription of *gusA* from P_*milA2*_, P_*milR*_ and P_*milA1*_ was not detected. These results indicated that the promoters of *milA4*-*E* operon, *orf1* and *milF*, are probably the direct targets of MilR.

### MilR activates the transcription of the *mil* gene cluster

**Fig. 4:**
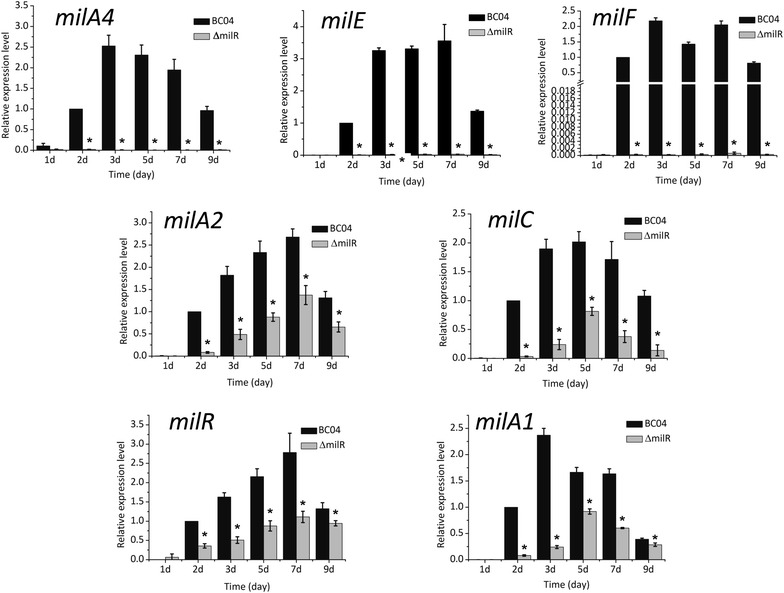
Quantitative real-time RT-PCR transcription profile analysis of *milA4*, *milE*, *milF*, *milA2*, *milC*, *milR* and *milA1*. All RNA samples were isolated from 1, 2, 3, 5, 7 and 9 days cultures. Data are presented as the averages of three independent experiments conducted in triplicate. 16S rRNA transcription was monitored and used as the internal control. *Error bars* show standard deviations, *P < 0.05

To determine the effect of *milR* on its target genes, quantitative real-time RT-PCR (qRT-PCR) analysis was performed to assess the transcriptional levels of *milA4*, *milE*, and *milF*. RNAs were prepared from cultures of BC04 and ΔmilR grown at different time points (1, 2, 3, 5, 7 and 9 days). These RNA samples were then subjected to qRT-PCR analysis. The results showed that transcripts of *milA4*, *milE* and *milF* were almost undetectable in ΔmilR compared with BC04, indicating that MilR activates the transcription of *milA4*, *milE* and *milF* directly (Fig. 4). In addition, we detected the expression of *milA2*, *milC*, *milR* and *milA1*. Transcription of these four genes decreased in ΔmilR (Fig. 4). However, GUS assays did not detect any interaction of MilR with the *milA2*, *milR* or *milA1* promoters (Fig. 3), indicating an indirect activation on these genes exerted by MilR. The decreased transcriptional levels of *milA2*, *milC*, *milR* and *milA1* were probably due to some unknown signals repressing the *mil* gene expression to avoid unnecessary consumption of nucleoside triphosphates (NTPs), by perceiving the inability of milbemycin production. Note that transcription of *orf1*, another putative target of MilR, was also undetectable in ΔmilR (data not shown). *orf1* encodes a enoylreductase, but is supposed to be unnecessary for milbemycin A3/A4 production. Therefore, further research on the biological significance of *orf1* as a target of MilR will not be mentioned in this work.

### Importance of the conserved AAA domain for the transcriptional regulation of MilR

**Fig. 5:**
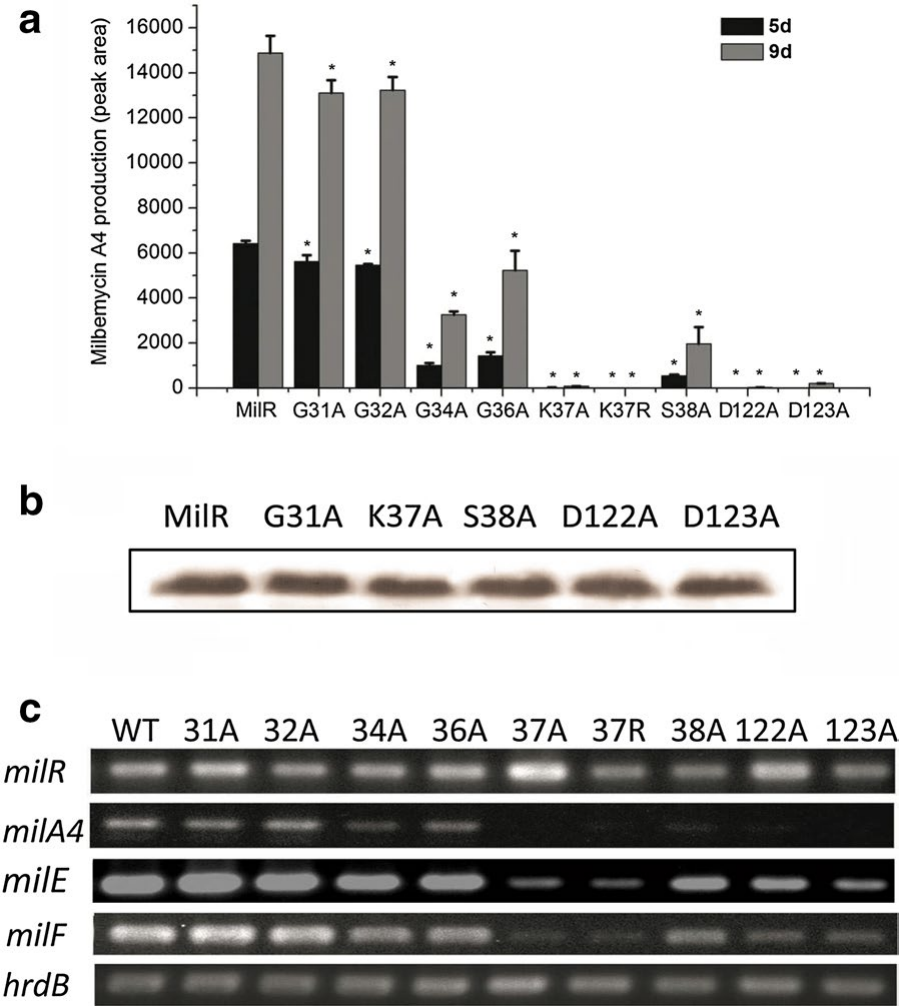
Effect of site-directed mutation in the AAA domain of MilR on its function. **a** Milbemycin production in the complementation strains containing the WT and mutant MilRs. MilR:ΔmilR/milR, G31A:ΔmilR/G31A, G32A:ΔmilR/G32A, G34A:ΔmilR/G34A, G36A:ΔmilR/G36A, K37A:ΔmilR/K37A, K37R:ΔmilR/K37R, S38A:ΔmilR/S38A, D122A:ΔmilR/D122A, D123A:ΔmilR/D123A. **b** Western blotting analysis of the WT and several selected mutant MilRs. The proteins of MilR and mutant MilRs (G31A, K37A, S38A, D122A and D123A) showed similar expression levels. **c** Transcriptional analysis of *milR*, *milA4*, *milE* and *milF* by semiquantitative RT-PCR. *hrdB* transcript was used as an internal control. MilR:ΔmilR/milR, 31A:ΔmilR/G31A, 32A:ΔmilR/G32A, 34A:ΔmilR/G34A, 36A:ΔmilR/G36A, 37A:ΔmilR/K37A, 37R:ΔmilR/K37R, 38A:ΔmilR/S38A, 122A:ΔmilR/D122A, 123A:ΔmilR/D123A; *P < 0.05

In MilR, the Walker A motif is GGPGCGKS and the Walker B motif is LVIAVDD. To define the relative contributions of Walker A and B to the function of MilR, site-directed mutations were introduced into certain conserved and less conserved residues in these two motifs (Additional file 3: Figure S3). We constructed pIJ10500::*milR*, pIJ10500::G31A, pIJ10500::G32A, pIJ10500::G34A, pIJ10500::G36A, pIJ10500::K37A, pIJ10500::K37R, pIJ10500::S38A, pIJ10500::D122A and pIJ10500::D123A. These integrative plasmids containing wild-type and mutant *milR*-coding nucleotide sequences were introduced into ΔmilR to obtain the complementary strains ΔmilR/MilR, ΔmilR/G31A, ΔmilR/G32A, ΔmilR/G34A, ΔmilR/G36A, ΔmilR/K37A, ΔmilR/K37R, ΔmilR/S38A, ΔmilR/D122A and ΔmilR/D123A, respectively. The strains above were verified by PCR (data not shown). Milbemycin production decreased in all strains containing the mutant MilR compared with ΔmilR/MilR (Fig. 5a), implying that the mutation of MilR seriously affected milbemycin production. Among them, ΔmilR/G37A, ΔmilR/G37R, ΔmilR/D122A and ΔmilR/D123A exhibited decreases of 98.5–99.5 %. ΔmilR/S38A decreased by 87 %, ΔmilR/G34A decreased by 78 %, ΔmilR/G36A decreased by 65 %, and ΔmilR/G31A and ΔmilR/G32A decreased by 15 %. These results showed that the functional importance of these amino acid residues is as follows: Lys37 ≈ Asp122 ≈ Asp123 > Ser38 > Gly34 > Gly36 > Gly31 ≈ Gly32.

To determine the effect of these mutant *milRs* on milbemycin biosynthetic genes, western blotting and RT-PCR analysis were carried out to assess the expression levels of *milR* and several structural genes. The wild-type and mutant *milR*s were transcribed and translated into proteins, as demonstrated by RT-PCR and western blotting (Fig. 5b, c), indicating that the site-directed mutagenesis did not influence the expression of MilR. In the mutants, except for ΔmilR/G31A and ΔmilR/G32A, the transcripts of *milA4*, *milE* and *milF* decreased substantially, whereas the transcript levels of *milA2*, *milC*, *milA3* and *milA1* were not affected significantly compared with that of ΔmilR/MilR (Additional file 4: Figure S4).These results strongly suggest that the Walker A and B sequences are essential for the transcriptional function of MilR.

### Enhancement of milbemycin A3/A4 production by overexpression of *milR*

**Fig. 6:**
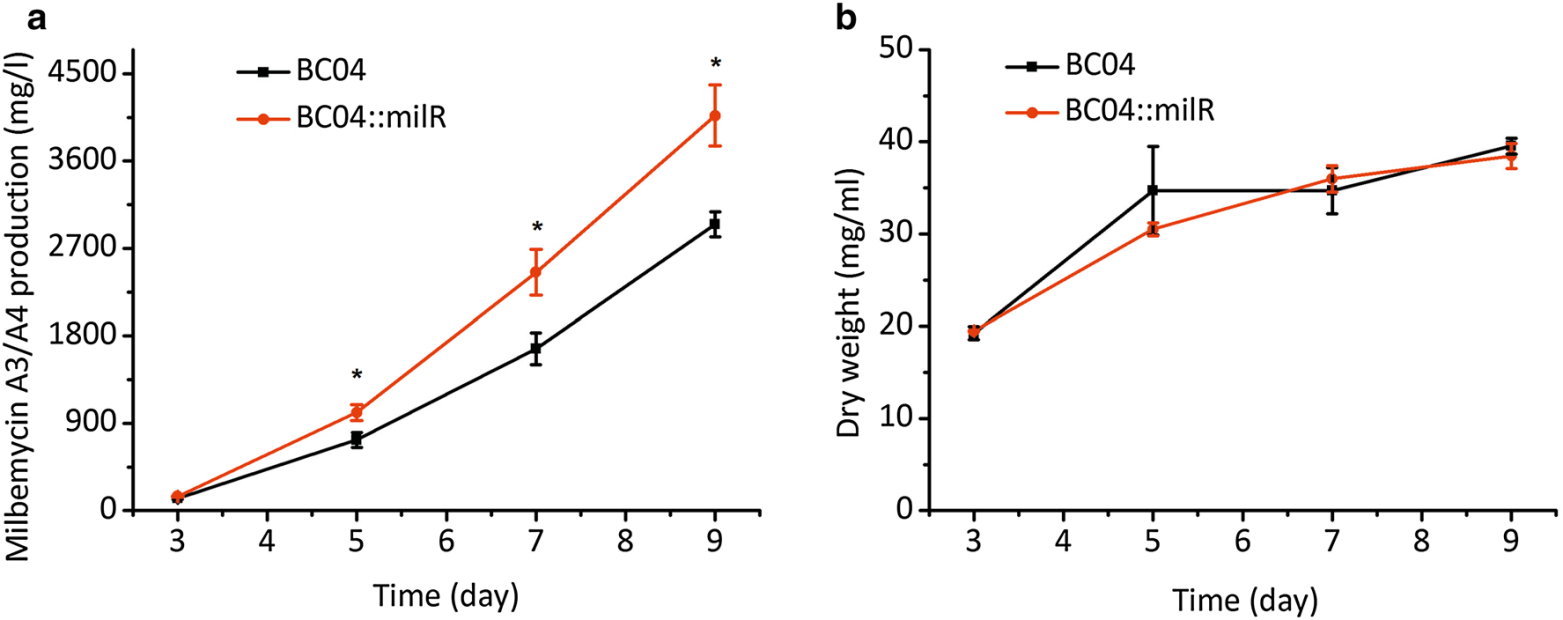
Effect of overexpression of *milR* on antibiotics production and cell dry weight. **a** Milbemycin production in *S. bingchenggensis* BC04 and BC04::milR. **b** Cell dry weight of *S. bingchenggensis* BC04 and BC04::milR. Data are presented as the averages of the results of three independent experiments. *Error bars* show standard deviations, *P < 0.05

Overexpression of transcriptional activators is an efficient approach to increase production of antibiotics, especially engineered activators under the control of strong constitutive promoters. Using this strategy, nikkomycin production in *Streptomyces ansochromogenes* and oxytetracycline biosynthesis in *Streptomyces rimosus* were enhanced significantly by introducing an extra copy of activator genes *sanG* (driven by constitutive *hrdB* promoter) and *otcR* (controlled by the constitutive SF14 promoter), respectively [30, 31]. In this work, *milR* was confirmed to be a direct activator of milbemycin biosynthesis (Fig. 2); thus, engineering the expression levels of *milR* might be a rational strategy to improve milbemycin production in *S. bingchenggensis* BC04, a high-producing industrial strain for milbemycin A3/A4 production. First, *milR* was placed under the control of a strong constitutive *hrdB* promoter in an integrative plasmid (pSET152) and in a multicopy plasmid (pKC1139). These two recombinant plasmids were transformed into BC04 to create BC04::hrdBmilR and BC04::hrdBmilRs, respectively. Unexpectedly, milbemycin A3/A4 production in BC04::hrdBmilR or BC04::hrdBmilRs was lower compared with that of BC04 (Additional file 5: Figure S5). Meanwhile, cell growth rate of these two strains was reduced in seed medium. BC04::hrdBmilRs exhibited a slower growth rate than that of BC04::hrdBmilR. These results indicated that overexpression of *milR* under a strong constitutive promoter did not increase milbemycin production. A single copy of *milR* with its own promoter was then cloned into pSET152 to generate pSET152::*milR*. This plasmid was transformed into BC04, resulting in BC04::milR. Notably, in BC04::milR, the final production of milbemycin A3/A4 increased by 38 %, reaching 4069 mg/l, compared with 2947 mg/l in BC04 (Fig. 6a). We also determined the biomass of BC04 and BC04::milR. The results showed that they had comparable growth rates and final biomass, and that the increase in milbemycin A3/A4 production was attributed to the overexpression of *milR* (Fig. 6b).

**Fig. 7:**
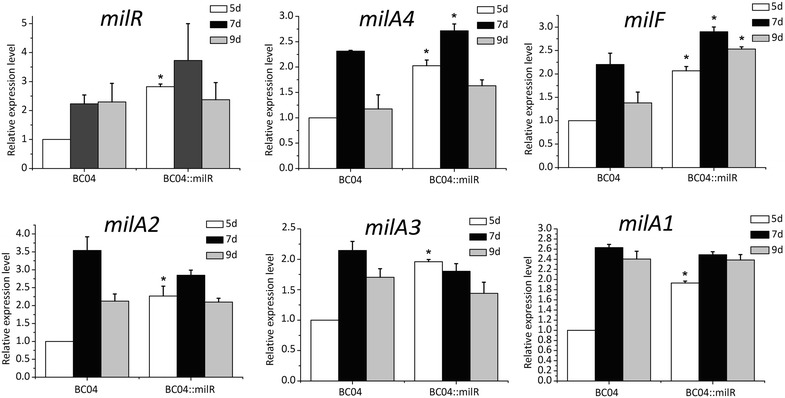
Quantitative real-time RT-PCR analysis of genes involved in milbemycin biosynthesis. The transcriptional levels of *mil* genes (*milR*, *milA4*, *milF*, *milA2*, *milA3* and *milA1*) are presented relative to that of BC04 sample collected after fermentation for 5 days, which was assigned a value of 1. Data are presented as the averages of the results of three independent experiments conducted in triplicate. 16S rRNA transcription was monitored and used as the internal control. *Error bars* show standard deviations, *P < 0.05

To verify whether the enhancement of milbemycin A3/A4 production was caused by the increased expression of *mil* genes, we measured the transcript levels of several *mil* genes in BC04 and BC04::milR. Transcription of *milR* was increased two*–*fold in BC04::milR over BC04 at 5 d, and remained higher than that in BC04 for several days thereafter (Fig. 7). MilR exerts its effect by activating the transcription of *milA4*-*E* and *milF*; therefore, the expression of *milA4* and *milF* was also detected (Fig. 7). As expected, the expressions of both *milA4* and *milF* were higher in BC04::milR than in BC04. Finally, based on the indirect activation by MilR on the *milA2*-*C*, *milR*-*A3* and *milA1*-*D* operons, transcript levels of *milA2*, *milA3* and *milA1* were assessed and shown to be increased two-fold at 5 days. These results showed that the overexpressed *milR* and the subsequent direct activation of *milA4*-*E*, *milF* and indirect activation of *milA2*-*C*, *milA3* and *milA1*-*D* accounted for the enhanced production of milbemycin A3/A4 in the engineered strain.

To further increase milbemycin A3/A4 production, a plasmid containing two copies of *milR* driven by its native promoter was constructed and introduced into BC04 to create BC04::2milR. However, the milbemycin A3/A4 production was less than that of BC04::milR (Additional file 5: Figure S5), accompanied by a decrease in cell growth in seed medium, indicating that excessive expression of *milR* does not coordinate the physiological conditions of the cell and lead to growth retardation.

## Discussion

Improving the titer of commercially valuable antibiotics is important for pharmaceutical industries to maintain sustainable development. Previously, random mutagenesis such as UV irradiation,* N*-methyl-*N*′-nitroso-*N*-nitrosoguanidine (NTG), atmospheric and room temperature plasma (ARTP) mutation, and rational metabolic engineering, have been carried out to improve milbemycin production in *S. bingchenggensis* [17, 18, 32]. However, a lack of knowledge about the regulation of milbemycin biosynthesis has meant that titer improvement of milbemycin production by manipulating regulatory genes has not been reported. In this study, a LAL activator gene, *milR*, the only regulator embedded within the milbemycin gene cluster, was identified, and its regulatory mechanism in milbemycin biosynthesis was characterized.

LAL regulators are widespread in *Streptomyces* with a varying number (from several to dozens), and mainly situated in clusters encoding type I polyketide synthases. To date, about 19 LALs have been reported to be cluster-situated activators in the regulation of antibiotic biosynthesis [19]. Among them, only the regulatory mechanisms of AveR in avermectin biosynthesis and PikD in pikomycin biosynthesis have been studied in any depth. PikD controls pikomycin biosynthesis by regulating two operons: the *pikAI*–*AIII* operon (comprising genes encoding polyketide chain initiation and elongation but not chain termination), and the *desI*–*V* operon (five co-transcribed genes encoding proteins responsible for desosamine biosynthesis) [24]. AveR controls the whole process of avermectin biosynthesis (including polyketide chain initiation, elongation and termination, polyketide modification, oleandrose biosynthesis and transglycosylation) by direct interaction with all *ave* promoters [21]. In the present study, we showed that MilR is a cluster-situated activator of milbemycin production, similar to the major LALs characterized in *Streptomyces*. The MilR sequence is highly similar to those of AveR and PikD, but exhibits a different method of regulation of milbemycin biosynthesis. In milbemycin biosynthesis, MilR controls the polyketide chain termination and modification specifically by directly activating the transcription of *milA4* (encoding the fourth PKS responsible for the final elongation and chain termination), *milE* and *milF*. In addition, MilR in *S. bingchenggensis* BC04 only affects milbemycin production and has no influence on polyether nanchangmycin production (another compound produced by *S. bingchenggensis*), while AveR in *S. avermitilis* not only activates avermectin production, but also cross-regulates oligomycin biosynthesis by directly repressing certain *oli* genes (e.g. *olmA1* and *olmA4*) [21].

In this study, we also observed a decrease in *milA2*, *milC*, *milR* and *milA1* transcripts in ΔmilR; however these genes might not be the direct targets of MilR, as shown by GUS assays (Fig. 3). The onset and production levels of each antibiotic are controlled by a large and diverse set of regulatory proteins, some of which can respond to (bind) physiological signals (e.g., antibiotics produced by the host) [19]. There is growing evidence that endogenous antibiotics can act as this type of physiological signal. In this scenario, they control their own biosynthesis by modulating the DNA-binding activity of cluster-situated or global regulatory proteins in a feed-forward or feedback mechanism [33]. For example, in *Streptomyces coelicolor*, the “pseudo” GBL receptor, ScbR2, can respond to (bind) endogenous Act and Red, thereby causing indirect activation of Act and Red production [34]. In *Streptomyces venezuelae*, JadR2, another homolog of ScbR2, binds the endogenous antibiotic chloramphenicol, thus activating the transcription of the *cml* structural gene *cmlJ* [34]. Recently, in *Streptomyces globisporus*, AtrA, a highly conserved TetR-family global regulator, was observed to activate lidamycin production by binding to the promoter region of *sgcR1R2* (via two CSRs within the lidamycin biosynthetic gene cluster). AtrA could bind the lidamycin intermediate heptaene, leading to its dissociation from the *sgcR1R2* promoter [35]. Based on these examples, we speculate that some unknown signals (i.e., certain regulatory proteins), could repress the milbemycin structural genes directly, or indirectly via regulatory cascades. We hypothesized that in the process of milbemycin biosynthesis, milbemycins accumulate to a threshold, which is sensed by the repressors. The repressors then dissociate from their target promoters and release the expression of milbemycin structural genes for milbemycin biosynthesis. Meanwhile in ΔmilR, cells could not produce milbemycins; therefore, these repressors could repress the expression of milbemycin structural genes continuously, leading to the observed decreases in *milA2*, *milC*, *milR* and *milA1* transcripts. We do not know the identity of these repressors; perhaps some of them are homologs of the “pseudo” GBL receptor or AtrA, which will require further confirmation. The search for upstream regulatory genes of *milA2*-*C*, *milR* and *milA1* is an important subject that we are currently working on.

Deep research on the *Escherichia coli* LAL protein, MalT, indicated that the Walker A and B motifs (AAA domain) in LALs are thought to be responsible for ATP binding and hydrolysis cycles. These cycles drive the protein to cycle between an ADP-bound, resting form and an ATP-bound, active form, which oligomerizes and initiates gene expression [36, 37]. However, data on the significance of Walker A and B sequences in *Streptomyces* LAL functions are limited: only two amino acid residues (K38 and D138 in AAA domain) have been verified as essential for PikD activity. To further understand the correlation between Walker A and B sequences and LAL function, we took MilR as a representative, and performed nine point mutations to measure the relative contribution of Walker A and B sequences in detail (Additional file 3: Figure S3). The putative ATP-binding site K37 (positively charged) was changed to Ala or a similar charged Arg (positively charged), Mg^2+^ binding site S38, and four Glycines in Walker A sequence were mutated to Ala, separately. In addition, the two Asps (aspartic acids D122 and D123) in Walker B were also changed to Ala, separately. Then MilR and the nine mutants mentioned above were checked for milbemycin A3/A4 production and target gene transcription. The results showed that changing K37 to Ala or to a similar charged Arg destroyed both milbemycin A3/A4 production and the efficiencies of MilR transcriptional activation (Fig. 5a, c), which is consistent with the previous conclusion that the K37 (Lys37) residue in the Walker A sequence is crucial for transcriptional regulation of AAA-containing regulatory proteins [38, 39]. Likewise, mutation of D122 to Ala and D123 to Ala also led to the loss of MilR function, while in PikD, the second Asp of Walker B (corresponding to D123 in MilR) has no influence on PikD activity [24], indicating a slight functional difference in the same Walker B site between MilR and PikD. S38 also has a large contribution in MilR function. Besides K37, D122, D123 and S38, G31 and G36 are two other highly conserved amino acid residues in the AAA domain of LAL regulators: our results indicated that G36 is more important than G31 in MilR function, but less important than K37 (Fig. 5a, c). Finally, G32 and G34 are two less conserved residues in Walker A. Interestingly, G32 and G34 exert an obvious influence in MilR activity. Expression of intact MilR in *E. coli* failed despite much effort, and we were unable to assess whether each mutation influenced ATP-binding affinity, ATPase activity or the aggregation state of MilR using in vitro experiments. However, these mutation data confirmed the functional importance of Walker A and B in MilR, and further indicated that certain amino acid residues in the walker A and B sequences might be the optimal combination for MilR function and its association with the cellular physiological environment. In addition, these results extended our understanding of the importance of the AAA domain in LALs that are widespread in *Streptomyces*.

Overexpression of transcriptional activators is often associated with an increase in production of the corresponding antibiotics [30]. The optimal product formation phenotype is obtained when activators are engineered to express at the optimum level and time (an expression profile that correlates well with cell physiological conditions) [40, 41]. In this work, *milR* was overexpressed using both native and strong constitutive *hrdB* promoters. Our results showed that the native promoter had a beneficial effect on milbemycin production, but the constitutive *hrdB* promoter had no effect. Overexpression of *milR* by introducing an extra copy driven by its native promoter improved milbemycin A3/A4 production significantly by 38 % in BC04 (Fig. 6). However, introduction of *milR* under the control of a strong constitutive *hrdB* promoter in an integrative plasmid pSET152 or in a multicopy plasmid pKC1139 did not result in a further increase but rather a lower level of milbemycin A3/A4 production and a decrease in cell growth compared with BC04. An additional two copies of *milR* driven by its own promoter also led to a decrease in milbemycin A3/A4 production and cell growth compared with that of BC04::milR (Additional file 5: Figure S5). These results suggested that the expression increase gained by integrating one copy of *milR* may be enough to improve milbemycin A3/A4 production in BC04, and that excessive expression of *milR* or expressing *milR* at a much earlier stage of growth has adverse effects on milbemycin A3/A4 biosynthesis and cell growth. This was partially confirmed by research on OTC, where two copies of overexpressed *otcR* could confer the highest OTC production but three additional copies caused a burden to the cell and led to decreased antibiotic production [31]. As for milbemycin biosynthesis, one additional copy of *milR* might be a better strategy to improve antibiotic production.

## Conclusions

In this study, we provided the first report of the regulatory mechanism in milbemycin biosynthesis in *S. bingchenggensis* BC04. MilR was confirmed to be a pathway-specific activator of milbemycin production. Moreover, our investigations demonstrated that overexpression of one copy of *milR* is an effective strategy to increase milbemycin production in an industrial strain. This is of practical importance for future industrial applications to improve milbemycin production in *Streptomyces*.

## Methods

### Bacterial strains and growth conditions

*Streptomyces bingchenggensis* BC04 is a derivative of *S. bingchenggensis* CGMCC 1734 deposited at China General Microbiological Culture Collection (CGMCC). *Streptomyces bingchenggensis* BC04 is a high-level producer of milbemycin A3/A4 generated from *S. bingchenggensis* CGMCC 1734 after random mutagenesis. *S. coelicolor* 1146 was used for GUS assays. *Escherichia coli* TOP 10 was used as a general host strain for propagating plasmids. *Escherichia coli* ET12567 (pUZ8002) was used for transferring DNA from *E. coli* to *Streptomyces* by conjugation [42]. For conjugation from *E. coli* to *Streptomyces*, *Streptomyces* strains were grown on mannitol/soya (MS) agar at 28 °C [42]. For genomic DNA extraction and spore collection, *S. bingchenggensis* BC04 and its derivatives were grown in SSPY (1 % sucrose, 0.1 % skimmed milk powder, 0.35 % peptone, 0.5 % yeast extract, and 0.05 % K_2_HPO_4_·3H_2_O, pH 7.2) liquid medium or on SSPY agar at 28 °C [18]. For milbemycin production, spore suspensions were inoculated in liquid SSPY and incubated at 28 °C on a rotary shaker (250 rpm) for 46 h as the seed culture, and then 6 ml of seed cultures was transferred into 250-ml Erlenmeyer flasks containing 50 ml fermentation medium (8 % sucrose, 2 % soybean powder, 0.1 % skimmed milk powder, 0.3 % CaCO_3_, 0.1 % K_2_HPO_4_, and 0.01 % FeSO4·7H_2_O, pH 7.0) for milbemycin production. For GUS assays, *S. coelicolor* 1146 derivatives were grown on AS-1 (0.1 % yeast extract, 0.02 % l-alanine, 0.02 % l-arginine, 0.02 % l-asparagine, 0.5 % soluble starch, 0.25 % NaCl, and 1 % Na_2_SO_4_) agar.

### Plasmids and DNA manipulation

pBluescript KS (+) was used for routine DNA cloning. The kanamycin resistance gene (*neo*) was obtained from a recombinant plasmid pUC119::*neo*. The *E. coli*-*Streptomyces* shuttle plasmid pKC1139, which contains a temperature-sensitive origin of replication from pSG5 [43], was used to construct recombinant plasmids for gene disruption and overexpression. pSET152 and pIJ10500, which can integrate into the *Streptomyces* chromosome by site-specific recombination at the phage ФC31 or ФBT1 attachment site (*attB*) respectively [43, 44], were used to create recombinant plasmids for introducing *milR* into *Streptomyces*. pIJ10500 also contains 3× FLAG tag, and was used in western blotting experiment. Plasmids and genomic DNA were isolated according to standard techniques [42]. Conjugal transfer from *E. coli* ET12567 (pUZ8002) into *S. bingchenggensis* was carried out as described previously [42].

### Sequence analysis

Protein sequence alignment and domain architectures were analyzed by using the BLAST (http://www.blast.ncbi.nlm.nih.gov/Blast.cgi), SMART (http://www.smart.embl-heidelberg.de/), Pfam (http://www.pfam.xfam.org/) and CDD (http://www.ncbi.nlm.nih.gov/cdd/) databases and software tool (Gene Doc).

### Construction of *milR* disruption mutant and its complementation

Disruption of *milR* was performed by gene replacement via homologous recombination. For construction of *milR* disruption mutant, two 2.2-kb fragments corresponding to the upstream and downstream sequences of *milR* were amplified from the *S. bingchenggensis* BC04 genomic DNA by polymerase chain reaction (PCR) using RD-LF/R and RD-RF/R as primer pairs (Additional file 6: Table S1). The upstream fragment was digested with *Eco*RI and *Kpn*I, and the downstream fragment was digested with *Bam*HI and *Xba*I. The kanamycin-resistance gene (*neo*) was obtained from pUC119::*neo* after digestion with *Kpn*I and *Bam*HI. These three resulting DNA fragments were ligated between the *Eco*RI and *Xba*I sites of pKC1139 to give pKC1139::*milR::neo*, in which a 1753-bp fragment of *milR* was replaced by *neo*. pKC1139*::milR::neo* was subsequently introduced into *S. bingchenggensis* BC04 via conjugation. Spores were harvested and spread on MS agar containing kanamycin. After incubation at 37 °C for 8 days, kanamycin resistant (Kan^R^) and apramycin sensitive (Apr^S^) colonies were identified, and further confirmed as *milR* disruption mutant (ΔmilR) by PCR.

For the complementation of *milR* in ΔmilR, a 3278-bp DNA fragment containing the coding region of *milR* and its upstream region was amplified using RCOMF and RCOMR (Additional file 6: Table S1) as primers, and then inserted between the *Eco*RV and *Xba*I sites of pSET152 to obtain pSET152::*milR*. Introduction of pSET152::*milR* into ΔmilR and the empty vector pSET152 into *S. bingchenggensis* BC04 by conjugation resulted in the complemented strain ΔmilR/pSET152::*milR* and the control strain BC04/pSET152, respectively.

### HPLC analysis of milbemycin A3/A4

The HPLC conditions for the detection of milbemycins were the same as previous report [18]. HPLC was performed with a Shimadzu LC-2010 CHT system (Shimadzu, Koyoto, Japan) by using a NOVA-PAK^R^ C18 column (3.9 × 150 mm, 5 μm, Waters) at a flow rate of 1.0 ml/min with a linear gradient from 0 to 100 % of solvent B in 15 min (Solvent A: CH_3_CN–H_2_O–CH_3_OH (350:50:100, v/v/v); Solvent B: CH_3_OH) and detected at 242 nm.

### Construction of the *gusA* reporter systems and GUS assays

A 1818-bp DNA fragment containing the complete *gusA* coding region was amplified from pIJ10500::*gusA* by PCR using gusA-F and gusA-R as primers (Additional file 1: Table S1). The six promoters of *milA2*, *milA4, milR*, *milA1*, *orf1* and *milF* were obtained from the genomic DNA of *S. bingchenggensis* BC04 by PCR with primer pair milA2-pF/pR, milA4-pF/pR, milF-pF/pR, orf1-pF/pR, milR-pF/pR and milA1-pF/pR, respectively (Additional file 6: Table S1). Prior to PCR amplification, primers gusA-F, milA2-pR, milA4-pR, milF-pR, orf1-pR, milR-pR and milA1-pR were phosphorylated with T4 polynucleotide kinase to facilitate subsequent ligation reactions. P_*milA2*_, P_*milA4*_, P_*milF*_, P_*orf1*_, P_*milR*_ and P_*milA1*_ were cut with *Xba*I and the *gusA* coding region was cut with *Eco*RI. The promoters and the *gusA* coding region were ligated together with *Xba*I/*Eco*RI double digested pSET152 in a three-piece ligation reaction to generate pSET152::P_*milA2*_*gusA*, pSET152::P_*milA4*_*gusA*, pSET152::P_*orf1*_*gusA*, pSET152::P_*milF*_*gusA*, pSET152::P_*milR*_*gusA* and pSET152::P_*milA1*_*gusA*. The authenticity of all PCR amplicons was verified by sequencing. After restriction digestion analysis, these pSET152-derived plasmids were integrated into the ФC31 *attB* site of *S. coelicolor* M1146 after conjugation via the donor strain *E. coli* ET12567/pUZ8002. Exconjugants were selected using apramycin and confirmed by PCR amplifications. These strains were used as negative controls in β-glucuronidase (GUS) assays and as conjugation recipients for construct in which MilR was expressed from the constitutive promoter P_*hrdB*_. For the construction of the latter construct, the *hrdB* promoter (P_*hrdB*_) was amplified with primer pair hrdB-pF and hrdB-pR, the *milR* coding region was amplified with primer pair milR-F and milR-R (Additional file 6: Table S1). Prior to PCR amplification, primers hrdB-pR and milR-F were phosphorylated with T4 polynucleotide kinase to facilitate subsequent ligation reactions. The *milR* coding region and P_*hrdB*_ digested with *Spe*I were ligated together with *Spe*I/*Stu*I double digested pIJ10500 in a three-piece ligation reaction to generate pIJ10500::P_*hrdB*_*milR*. Exconjugants were selected using hygromycin and further confirmed by PCR amplifications. For GUS assays, spores of *S. coelicolor* 1146 derived strains were harvested, resuspended in double-distilled water (ddH_2_O), and optical density at 450 nm was measured. The spore suspension was normalized to the same level, series diluted, and spotted on 25-ml plates of AS-1 agar containing 100 μl of 40 mg/ml 5-bromo-4-chloro-3-indolyl-β-D-glucoronide and photographed after 3 days at 28 °C [45, 46]. For GUS assay of *milA4*-*E* promoter, the plate of AS-1 agar containing chromogenic substrate was photographed after 7 days cultivation at 28 °C.

### RNA isolation, RT-PCR and quantitative real-time RT-PCR

RNAs were isolated from *S. bingchenggensis* BC04 grown at 28 °C at different time points (1, 2, 3, 5, 7 and 9 days). The detailed steps for RNA extraction were described previously [47]. To exclude the possibility of genomic DNA contamination, each RNA sample was treated with RQ1 RNase-free DNase I (Promega). The quality and quantity of RNAs were examined by UV spectroscopy and agarose gel electrophoresis. For RT-PCR and quantitative real time RT-PCR, first-strand cDNA synthesis was carried out with the superscript III first-strand synthesis system (Invitrogen, California, USA) using 1 μg total RNAs following the manufacturer’s instructions. All cDNA synthesis reactions included a replicate reaction without reverse transcriptase to ensure the complete removal of contaminating DNA from the RNA samples. For RT-PCR, after 31 cycles of amplification, the products were examined by the 1.5 % agarose gel electrophoresis and then visualized by staining with ethidium bromide. For quantitative real time RT-PCR, oligonucleotides were designed to amplify fragments of 100-200 bp (Additional file 6: Table S1). The PCR procedures were as follows: reactions were performed in 96-well plates using a ROCHE LightCycler®-96. Each 20 μl reaction contained 10 μl of 2× SuperReal PreMix (SYBR Green I included), 6 pmol of each primer and 1 μl five-fold diluted cDNA. The reaction parameters were as follows: 95 °C for 10 min, followed by 40 three-step amplification cycles consisting of denaturation at 95 °C for 15 s, annealing at 60 °C for 60 s and extension at 72 °C for 30 s. A final dissociation stage was run to generate a melting curve and consequently verify the specificity of the amplification products. After the PCR amplifications, data were analyzed by LightCycler®-96 Series Software. All samples were run in triplicate. The transcriptional level of target genes was normalized internally to the level to 16S rRNA transcription according to the Livak’s method [48].

### Western blotting of MilR and its derivatives in *S. bignchenggensis*

For western blotting analysis, cell extracts from ΔmilR/MilR, ΔmilR/G31A, ΔmilR/G37A, ΔmilR/G38A, ΔmilR/D122A and ΔmilR/D123A grown in the fermentation medium at 3 days, were sonicated on ice. The concentration of total protein was determined by BCA protein assay using BSA as the standard sample. Proteins with equal concentrations (80 μg) samples were loaded onto 12 % polyacrylamide/SDS gel electrophoresis. Proteins in the gels were transferred to PVDF western blotting membranes (Roche, Germany) and probed with monoclonal ANTI-FLAGM2 antibody (Sigma-Aldrich, USA) as recommended by the manufacturer. The antibodies on the membranes were hybridized with the Horse Anti-Mouse IgG (H + L)-AP as secondary antibody (ZSGB-BIO, China) and the position of tagged-FLAG was visualized through BCIP/NBT method.

### Site-directed mutagenesis of *milR*

For construction of wild-type *milR* complement plasmid, a 3278-bp DNA fragment containing the complete *milR* coding region and its promoter region was amplified using RCOMF2 and RCOMR2 (Additional file 6: Table S1) as primers, and then cloned between the *Spe*I and *Stu*I sites of pIJ10500 to obtain pIJ10500::*milR*. Meanwhile the 3278-bp fragment was also inserted into the *Eco*RV site of pBluescriptKS (+) to generate pBluescriptKS (+)::*milR*, which was used as a template plasmid. For the construction of mutant *milR*, the primer pairs (Additional file 6: Table S1) were phosphorylated at the 5′ end with T4 polynucleotide kinase, and were further used for amplication of mutant *milR* fragment by PCR from the template plasmid pBluescriptKS (+):: *milR.* The PCR products were purified by gel extraction and ligated by self-ligation using T4 DNA ligase to generate mutagenized *milR* plasmids. The mutants of *milR* are as follows: (1) G31A (GGC to GCG), (2) G32A (GGG to GCG), (3) G34A (GGG to GCG), (4) G36A (GGC to GCG), (5) K37A (AAG to GCG), (6) S38A (AGC to GCG), (7) D122A (GAC to GCG), (8) D123A (GAT to GCG) and (9) K37R (AAG to CGC). These mutated plasmids were then digested with *Spe*I and *Ahd*I respectively, giving a 721-bp restriction fragment that contains the mutated site inside *milR*. The *Spe*I–*Ahd*I fragments were then cloned between the *Spe*I and *Ahd*I sites of the pIJ10500::*milR* to generate pIJ10500::G31A, pIJ10500::G32A, pIJ10500::G34A, pIJ10500::G36A, pIJ10500::K37A, pIJ10500::K37R, pIJ10500::S38A, pIJ10500::D122A and pIJ10500::D123A. The authenticity of the whole sequence of *milR* was verified by sequencing. Subsequently, pIJ10500::*milR* and these pIJ10500::*milR*-derived plasmids were then integrated into the chromosomal ФBT1 *attB* site of *milR* disruption mutant (ΔmilR) by conjugation.

### Construction of *milR* overexpression strains in *S. bingchenggensis* BC04

For the construction of pSET152::P_*hrdB*_*milR* and pKC1139::P_*hrdB*_*milR*, the *hrdB* promoter (P_*hrdB*_) was amplified with primer pair hrdB-pF and hrdB-pR, and the *milR* coding region was amplified with primer pair milR-F and milR-R (Additional file 6: Table S1). Prior to PCR amplification, primers hrdB-pR and milR-F were phosphorylated with T4 polynucleotide kinase to facilitate subsequent ligation reactions. P_*hrdB*_ digested with *Xba*I and the *milR* coding region were ligated together with *Xba*I/*Eco*RV double digested pSET152 and pKC1139 in a three-piece ligation reaction to generate pSET152::P_*hrdB*_*milR* and pKC1139::P_*hrdB*_*milR*. For the construction of pSET152::2*milR*, two 3278-bp DNA fragments containing the complete *milR* coding region and its promoter region were amplified with primer pairs ROE-F/ROE-R1 and ROE-F1/ROE-R (Additional file 6: Table S1). The fragment amplified with ROE-F/ROE-R1 was digested with *Xba*I and *Bam*HI, another fragment amplified with ROE-F1/ROE-R was digested with *Bam*HI. Then the two digested fragments were ligated together with *Xba*I/*Eco*RV double digested pSET152 in a three-piece ligation reaction to generate pSET152::2*milR*. These three plasmids (pSET152::P_*hrdB*_*milR*, pKC1139::P_*hrdB*_*milR* and pSET152::2*milR*) together with pSET152::*milR* were introduced into *S. bingchenggensis* BC04 by conjugation and resulted in the overexpressed strain BC04::hrdBmilR, BC04::hrdBmilRs, BC04::milR and BC04::2milR.

### Determination of cell dry weight

Five-milliliter cell cultures were collected by vacuum filtration and dried at 60 °C to a constant weight.

### Statistical analysis

All experiments were carried out independently at least three times, and the mean values ±SD were presented. The data were analyzed by Student’s t test. *P* < 0.05 is used as a standard criterion of statistical significance.

## Additional files

10.1186/s12934-016-0552-1 Confirmation of *milR* disruption by PCR amplification. A. The schematic diagram showing the position of primers in the chromosome of double-crossover strains. Among the two primer pairs used for PCR, one primer (conR1F or conR2R) in each pair is designed outside of the upstream (L) or downstream (R) sequence we used for construction of *milR* disruption mutant. B. Agarose gel electrophoresis showing PCR amplified fragments. PCR templates were genomic DNAs from the *Streptomyces bingchenggensis* BC04 (lane 1) and the three independent mutants as indicated (lanes 2–4). The primer pairs used are also shown. Then theoretical size of DNA fragments (4.5-kb and 3.5-kb) for ΔmilR were obtained by PCR, and further confirmed by DNA sequencing (data not shown).
10.1186/s12934-016-0552-1 Co-transcriptional analysis of the biosynthetic genes in *mil* cluster. **A.** Intergenic regions numbered 1-4 were subjected to RT-PCR analysis and the occurrence of translational coupling between *milA1* and *milD* were marked by “▲”, this location was not subjected to RT-PCT analysis. **B.** RT-PCR analysis of co-transcribed genes. Primers were designed to amplify 4 intergenic regions (numbered 1-4, Table S1). cDNA was synthesized from RNA samples isolated from *S. bingchenggensis* BC04 cultures (3 d cultivation in fermentation medium). Positive controls: PCR reactions using genomic DNA of BC04 as template which gave products of the same molecular sizes as those amplified from cDNA template obtained by reverse transcription. Negative controls: PCR amplification of *hrdB* using “cDNA” template synthesized without reverse transcriptase, which did not give products, thus excluding the possible contamination of genomic DNA. c: cDNA template; g: genomic DNA template; NRT: “cDNA” template synthesized in the absence of reverse transcriptase. These results show that *milA2* and *milC*, *milA4* and *milE*, *milR* and *milA3*, *milA1* and *milD* form an operon respectively, whereas the other two genes *milF* and *orf1* are transcribed individually.
10.1186/s12934-016-0552-1 Diagrams of site-directed mutation of Walker A and Walker B motifs in MilR. **A:** Mutation in Walker A motif. The first line shows the wild-type Walker A sequence. From the second to the eighth line, red words indicate the Ala or Arg substitution was performed in the corresponding position. **B:** Mutation in Walker B motif. The first line shows the wild-type Walker B sequence, from the second to the third line, blue words indicate the Ala substitution was carried out to replace Asp in the corresponding position.
10.1186/s12934-016-0552-1 Effect of site-directed mutation in the AAA domain of MilR on the transcription of *milA2*, *milC*, *milA3* and *milA1*. Transcriptional analysis of *milA2*, *milC*, *milA3* and *milA1* were performed by semiquantitative RT-PCR. MilR:ΔmilR/milR, 31A:ΔmilR/G31A, 32A:ΔmilR/G32A, 34A:ΔmilR/G34A, 36A:ΔmilR/G36A, 37A:ΔmilR/K37A, 37R:ΔmilR/K37R, 38A:ΔmilR/S38A, 122A:ΔmilR/D122A, 123A:ΔmilR/D123A.
10.1186/s12934-016-0552-1 Effect of *milR* engineering on antibiotic production. Data are presented as the averages of the results of three independent experiments. Error bars show standard deviations.
10.1186/s12934-016-0552-1 Primers used in this study.

## Abbreviations

AAA: ATPases associated with diverse cellular activities
Ala (A): alanine
ARTP: atmospheric and room temperature plasma
CDD: conserved domain database
CSR: cluster-situated regulator
D: Asp, aspartic acid
G: Gly, glycine
GBL: γ-butyrolactones
GUS: β-glucuronidase
HPLC: high performance liquid chromatography
HTH-LuxR: helix_turn_helix, luminescence regulator
K: Lys, lysine
LAL: large ATP-binding regulator of the LuxR family
MS: mannitol/soya
NTG: N-methyl-N′-nitroso-N-nitrosoguanidine
NTPases: nucleoside triphosphatases
NTPs: nucleoside triphosphates
ORF: open reading frame
OTC: oxytetracycline
PCR: polymerase chain reaction
PKS: polyketide synthase
qRT-PCR: quantitative real-time RT-PCR
R: Arg, arginine
RT-PCR: reverse transcription polymerase chain reaction
S: Ser, serine
SMART: simple modular architecture research tool
