# Supplementary material for: Characterization of a pathway-specific activator of milbemycin biosynthesis and improved milbemycin production by its overexpression in Streptomyces bingchenggensis
Source: Microb Cell Fact. 2016 Sep 7;15(1):152. doi: 10.1186/s12934-016-0552-1 (PMC5015266; doi:10.1186/s12934-016-0552-1)
Supplement: Supplementary file 1 — 10.1186/s12934-016-0552-1 Confirmation of milR disruption by PCR amplification. A. The schematic diagram showing the position of primers in the chromosome of double-crossover strains. Among the two primer pairs used for PCR, one primer (conR1F or conR2R) in each pair is designed outside of the upstream (L) or downstream (R) sequence we used for construction of milR disruption mutant. B. Agarose gel electrophoresis showing PCR amplified fragments. PCR templates were genomic DNAs from the Streptomyces bingchenggensis BC04 (lane 1) and the three independent mutants as indicated (lanes 2–4). The primer pairs used are also shown. Then theoretical size of DNA fragments (4.5-kb and 3.5-kb) for ΔmilR were obtained by PCR, and further confirmed by DNA sequencing (data not shown). [file 12934_2016_552_MOESM1_ESM.pdf]

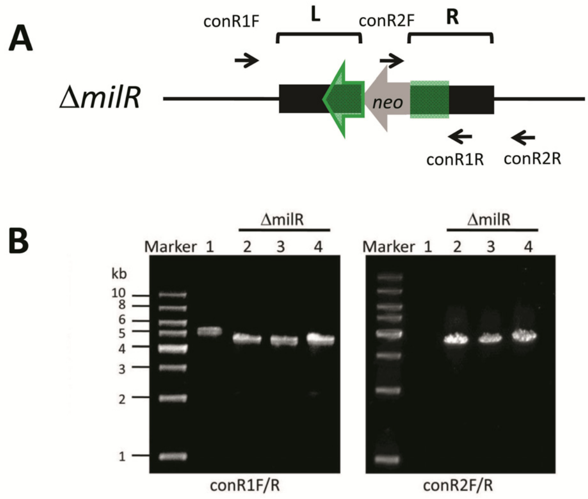

Figure S1 Confirmation of *milR* disruption by PCR amplification.

A. The schematic diagram showing the position of primers in the chromosome of double-crossover strains. Among the two primer pairs used for PCR, one primer (conR1F or conR2R) in each pair is designed outside of the upstream (L) or downstream (R) sequence we used for construction of *milR* disruption mutant.

B. Agarose gel electrophoresis showing PCR amplified fragments. PCR templates were genomic DNAs from the *Streptomyces bingchenggensis* BC04 (lane 1) and the three independent mutants as indicated (lanes 2–4). The primer pairs used are also shown. Then theoretical size of DNA fragments (4.5-kb and 3.5-kb) for ΔmilR were obtained by PCR, and further confirmed by DNA sequencing (data not shown).
