# Supplementary material for: Characterization of a pathway-specific activator of milbemycin biosynthesis and improved milbemycin production by its overexpression in Streptomyces bingchenggensis
Source: Microb Cell Fact. 2016 Sep 7;15(1):152. doi: 10.1186/s12934-016-0552-1 (PMC5015266; doi:10.1186/s12934-016-0552-1)
Supplement: Supplementary file 2 — 10.1186/s12934-016-0552-1 Co-transcriptional analysis of the biosynthetic genes in mil cluster. A. Intergenic regions numbered 1-4 were subjected to RT-PCR analysis and the occurrence of translational coupling between milA1 and milD were marked by “▲”, this location was not subjected to RT-PCT analysis. B. RT-PCR analysis of co-transcribed genes. Primers were designed to amplify 4 intergenic regions (numbered 1-4, Table S1). cDNA was synthesized from RNA samples isolated from S. bingchenggensis BC04 cultures (3 d cultivation in fermentation medium). Positive controls: PCR reactions using genomic DNA of BC04 as template which gave products of the same molecular sizes as those amplified from cDNA template obtained by reverse transcription. Negative controls: PCR amplification of hrdB using “cDNA” template synthesized without reverse transcriptase, which did not give products, thus excluding the possible contamination of genomic DNA. c: cDNA template; g: genomic DNA template; NRT: “cDNA” template synthesized in the absence of reverse transcriptase. These results show that milA2 and milC, milA4 and milE, milR and milA3, milA1 and milD form an operon respectively, whereas the other two genes milF and orf1 are transcribed individually. [file 12934_2016_552_MOESM2_ESM.pdf]

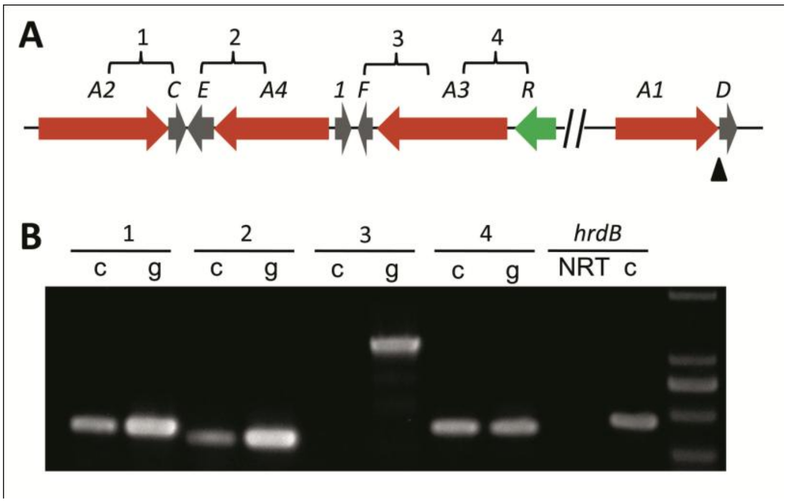

Figure S2 Co-transcriptional analysis of the biosynthetic genes in *mil* cluster.

A. Intergenic regions numbered 1-4 were subjected to RT-PCR analysis and the occurrence of translational coupling between *milA1* and *milD* were marked by "▲", this location was not subjected to RT-PCT analysis. **B.** RT-PCR analysis of co-transcribed genes. Primers were designed to amplify 4 intergenic regions (numbered 1-4, Table S1). cDNA was synthesized from RNA samples isolated from *S. bingchenggensis* BC04 cultures (3 d cultivation in fermentation medium). Positive controls: PCR reactions using genomic DNA of BC04 as template which gave products of the same molecular sizes as those amplified from cDNA template obtained by reverse transcription. Negative controls: PCR amplification of *hrdB* using "cDNA" template synthesized without reverse transcriptase, which did not give products, thus excluding the possible contamination of genomic DNA. c: cDNA template; g: genomic DNA template; NRT: "cDNA" template synthesized in the absence of reverse transcriptase. These results show that *milA2* and *milC*, *milA4* and *milE*, *milR* and *milA3*, *milA1* and *milD* form an operon respectively, whereas the other two genes *milF* and *orf1* are transcribed individually.
