# Supplementary material for: Characterization of a pathway-specific activator of milbemycin biosynthesis and improved milbemycin production by its overexpression in Streptomyces bingchenggensis
Source: Microb Cell Fact. 2016 Sep 7;15(1):152. doi: 10.1186/s12934-016-0552-1 (PMC5015266; doi:10.1186/s12934-016-0552-1)
Supplement: Supplementary file 3 — 10.1186/s12934-016-0552-1 Diagrams of site-directed mutation of Walker A and Walker B motifs in MilR. A: Mutation in Walker A motif. The first line shows the wild-type Walker A sequence. From the second to the eighth line, red words indicate the Ala or Arg substitution was performed in the corresponding position. B: Mutation in Walker B motif. The first line shows the wild-type Walker B sequence, from the second to the third line, blue words indicate the Ala substitution was carried out to replace Asp in the corresponding position. [file 12934_2016_552_MOESM3_ESM.pdf]

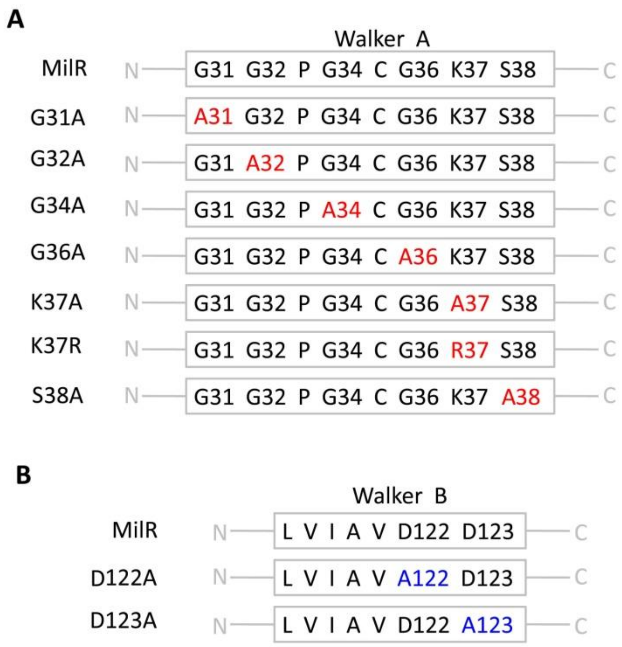

Figure S3 Diagrams of site-directed mutation of Walker A and Walker B motifs in MilR

A: Mutation in Walker A motif. The first line shows the wild-type Walker A sequence. From the second to the eighth line, red words indicate the Ala or Arg substitution was performed in the corresponding position.

B: Mutation in Walker B motif. The first line shows the wild-type Walker B sequence, from the second to the third line, blue words indicate the Ala substitution was carried out to replace Asp in the corresponding position.
