# Supplementary material for: Characterization of a pathway-specific activator of milbemycin biosynthesis and improved milbemycin production by its overexpression in Streptomyces bingchenggensis
Source: Microb Cell Fact. 2016 Sep 7;15(1):152. doi: 10.1186/s12934-016-0552-1 (PMC5015266; doi:10.1186/s12934-016-0552-1)
Supplement: Supplementary file 4 — 10.1186/s12934-016-0552-1 Effect of site-directed mutation in the AAA domain of MilR on the transcription of milA2, milC, milA3 and milA1. Transcriptional analysis of milA2, milC, milA3 and milA1 were performed by semiquantitative RT-PCR. MilR:ΔmilR/milR, 31A:ΔmilR/G31A, 32A:ΔmilR/G32A, 34A:ΔmilR/G34A, 36A:ΔmilR/G36A, 37A:ΔmilR/K37A, 37R:ΔmilR/K37R, 38A:ΔmilR/S38A, 122A:ΔmilR/D122A, 123A:ΔmilR/D123A. [file 12934_2016_552_MOESM4_ESM.pdf]

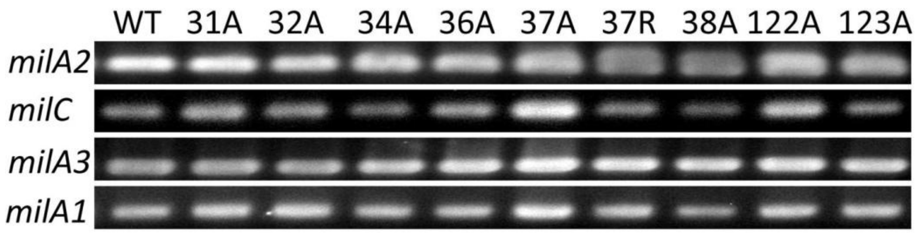

Figure S4 Effect of site-directed mutation in the AAA domain of MilR on the transcription of *milA2*, *milC*, *milA3* and *milA1*.

Transcriptional analysis of *milA2*, *milC*, *milA3* and *milA1* were performed by semiquantitative RT-PCR. MilR:ΔmilR/milR, 31A:ΔmilR/G31A, 32A:ΔmilR/G32A, 34A:ΔmilR/G34A, 36A:ΔmilR/G36A, 37A:ΔmilR/K37A, 37R:ΔmilR/K37R, 38A:ΔmilR/S38A, 122A:ΔmilR/D122A, 123A:ΔmilR/D123A.
