# Supplementary material for: Characterization of a pathway-specific activator of milbemycin biosynthesis and improved milbemycin production by its overexpression in Streptomyces bingchenggensis
Source: Microb Cell Fact. 2016 Sep 7;15(1):152. doi: 10.1186/s12934-016-0552-1 (PMC5015266; doi:10.1186/s12934-016-0552-1)
Supplement: Supplementary file 5 — 10.1186/s12934-016-0552-1 Effect of milR engineering on antibiotic production. Data are presented as the averages of the results of three independent experiments. Error bars show standard deviations. [file 12934_2016_552_MOESM5_ESM.pdf]

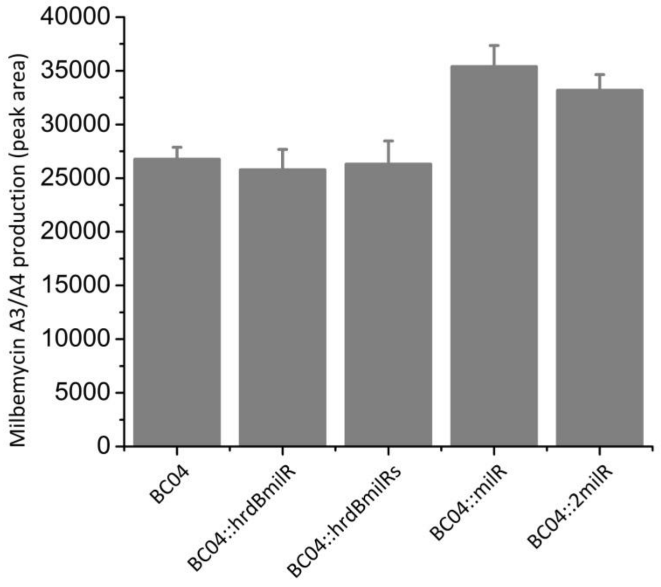

Figure S5 Effect of *milR* engineering on antibiotic production. Data are presented as the averages of the results of three independent experiments. Error bars show standard deviations
