# Supplementary material for: Characterization of a pathway-specific activator of milbemycin biosynthesis and improved milbemycin production by its overexpression in Streptomyces bingchenggensis
Source: Microb Cell Fact. 2016 Sep 7;15(1):152. doi: 10.1186/s12934-016-0552-1 (PMC5015266; doi:10.1186/s12934-016-0552-1)
Supplement: Supplementary file 6 — 10.1186/s12934-016-0552-1 Primers used in this study. [file 12934_2016_552_MOESM6_ESM.docx]

**Table S1. Primers used in this study**

| **Primer** | **Sequence (5’-3’)** |
| --- | --- |
| **For disruption and complementation** | |
| RD-LF | CGGAATTCCAGCGACACCATCACCGAGAACA |
| RD-LR | CGGGGTACCGACTGAGCGAGTCGGAAATGCGT |
| RD-RF | CGGGATCCCAGCAGTCGGCTCAGCAACG |
| RD-RR | GCTCTAGACTCGAAATCCTTCTGGGTCAGGT |
| RCOMF (ROE-F) | GCTCTAGACGAGACCGTCCCTATGTCCGATG |
| RCOMR (ROE-R) | ACATATGCAACCTGTGGACCTCCGTGGCA |
| **For confirmation of disrupted mutants** | |
| conR1F | GCTCCACGGCTTCGATATGCG |
| conR1R | ATGCTCATTGACCGGAGCGCTCAC  [Main Instruction]  HP LaserJet Service 已停止工作  [Content]  Windows 正在检查该问题的解决方案...  [取消] |
| conR2F | AATGAATTCGAGCTCGGTACCCGAAC |
| conR2R | CGGCAATAGGTGATTCCAGGACAAC |
| **For analysis of RT-PCR** | |
| milA2-CF | TCGGACGCAGGCGAGGAGGA |
| milA2-CR | GCCCACAGTGTTACGGGAAGGGT |
| milA4-EF | TGCGACCGAGCCGGATTC |
| milA4-ER | CACTTCACCATGCTGGAGGAGC |
| milA3-FF | CCGCCCTGATGGCGAATGC |
| milA3-FR | TCGGTCACGGACGATGAGATGTT |
| milR-A3F | TACAGCCGCTCCACGTCCCA |
| milR-A3R | CCGCCAGCAACTGCCAAAG |
| RThrdBF | CCCGAGAACCAGGGCTTTGTG |
| RThrdBR | CCAGGTGGCGTAGGTGGAGAAC |
| RTmilA2F | GGTGGGAAGGGCAGTTGTGGG |
| RTmilA2R | TCGTCGGTCAGGGTCTGGATGG |
| RTmilCF | GGCTTTCTGCTCGTCGTGCTCC |
| RTmilCR | TGTAGAGGGCGATGCTGATGTTGG |
| RTmilA4F | AGGACCTTGAGGGCTATGCGATGAC |
| RTmilA4R | CGCTGCTGTGACGGACCATTCG |
| RTmilEF | CGCTCGACTTCCTGTCCTCCCTG |
| RTmilER | GACTTGGCGATCATTGCTGTGGC |
| RTmilFF | GCCGCCGAAGGTGTTCTGGTG |
| RTmilFR | CGCGATGGTGTTGACGGTGATTC |
| RTmilRF | TGAGCCGACTGCTGGGACTGG |
| RTmilRR | AGCGTGTCGCCGAGGTGGTG |
| RTmilA3F | ACCCACTACCACCCAAACCTCCC |
| RTmilA3R | GCCAGTTGCTCGTACAGTCCCTCC |
| RTmilA1F | GGCAACCGTCATCTCGGGAAACA |
| RTmilA1R | TCGTGGAGCCAGTAGTGGTGGTGTT |
| **For GUS assays** | |
| milA2-pF | GCTCTAGAACCCGGCCACCATCTCGG |
| milA2-pR | CAAAGTGGGATCACTTTCTTGAGCG |
| milA4-pF | GCTCTAGAGGCTACCGCCTCTCGGGAGGAG |
| milA4-pR | TTACGGCATCTCCTCGACATTCCTCA |
| orf1-pF | GCTCTAGACGCGGAGTGGCTGCGTGTACG |
| orf1-pR | TGCGAACTCGTTCTGGGCTTTGG |
| milF-pF | GCTCTAGACGCGGAGTGGCTGCGTGTACG |
| milF-pR | TGCGAACTCGTTCTGGGCTTTGG |
| milR-pF | GCTCTAGATTCTTGTCTCCCCTTGTTACGGCTGG |
| milR-pR | GGCCCTCCAGGACTGCTGTCACG |
| milA1-pF | GCTCTAGACGCGGAGTGGCTGCGTGTACG |
| milA1-pR | TGCGAACTCGTTCTGGGCTTTGG |
| gusA-F | ATGACCGGTCTGCGGCCCGTCG |
| gusA-R | CGGAATTCTCACTGCTTCCCGCCCTGCTGCGGC |
| hrdB-pF | AATTTCTAGACCGCCTTCCGCCGGAACG |
| hrdB-pR | GAACAACCTCTCGGAACGTTG |
| milR-F | ATGCTCATTGACCGGAGCGCTCAC |
| milR-R | CCTCTGCCGCTCGTTGGTCTTG |
| **For quantitative real-time RT-PCR analysis** | |
| real-A4F | TTGAGGGCTATGCGATGAC |
| real-A4R | ACGACTGACACGCCAAAT |
| real-EF | GTCATGTACAGCGCCTACG |
| real-ER | TTGTAGACACCTCCCGAGAA |
| real-FF | CGCTGAACATCCTGGTGAA |
| real-FR | CCGTACATTGACGCTGAAGA |
| real-A2F | CCGCAGGAATCAACCTGAA |
| real-A2R | GGCAATGGAGGAGAAGAGAAC |
| real-CF | GGTATCAGTTCCCGCTGTATC |
| real-CR | GAGGGCGATGCTGATGTT |
| real-RF | GCAAGAGCGAATTCCTGAAG |
| real-RR | CTGTGACGGAGCGAGTC |
| real-A1F | GCCGTATCGTCTTCGTCTTC |
| real-A1R | TGGAGGATGTCCGTGAGT |
| real-16sRNA-F | TGTCGTGAGATGTTGGGTTAAG |
| real-16sRNA-R | TCATTGTACCGGCCATTGTAG |
| **For site-directed mutagenesis of *milR*** | |
| RCOMF2 | GGACTAGTCGAGACCGTCCC TATGTCCGATG |
| RCOMR2 | GAGCGAGGACAGCTCTCTGCGGTC |
| Walker A-G31AF | ***GCG***GGGCCGGGGTGCGGCAAGAGCGAATTCCT |
| Walker A-G32AF | GGC***GCG***CCGGGGTGCGGCAAGAGCGAATTCCT |
| Walker A-G34AF | GGCGGGCCG***GCG***TGCGGCAAGAGCGAATTCCT |
| Walker A-G36AF | GGCGGGCCGGGGTGC***GCG***AAGAGCGAATTCCT |
| Walker A-K37AF | GGCGGGCCGGGGTGCGGC***GCG***AGCGAATTCCT |
| Walker A-K37RF | GGCGGGCCGGGGTGCGGC***CGC***AGCGAATTCCT |
| Walker A-S38AF | GGCGGGCCGGGGTGCGGCAAG***GCG***GAATTCCTGAAG |
| Walker A-MUTR | CTCGGCGACCAACAGCCGGAAATCGCCTGCCG |
| Walker B-D122AF | ***GCG***GATCTGCACCACGCGGACCAGGCGTCGCTC |
| Walker B-D123AF | GAC***GCG***CTGCACCACGCGGACCAGGCGTCGCTC |
| Walker B-MUTR | GACCGCGATCACCAGCGGTGCGGACGC |
| **For overexpression of MilR** | |
| ROE-F | GCTCTAGACGAGACCGTCCCTATGTCCGATG |
| ROE-R | ACATATGCAACCTGTGGACCTCCGTGGCA |
| hrdB-pF | AATTTCTAGACCGCCTTCCGCCGGAACG |
| hrdB-pR | GAACAACCTCTCGGAACGTTG |
| milR-F | ATGCTCATTGACCGGAGCGCTCAC |
| milR-R | CCTCTGCCGCTCGTTGGTCTTG |
| ROE-F1 | CGGGATCCCGAGACCGTCCCTATGTCCGATG |
| ROE-R1 | CGGGATCCCAACCTGTGGACCTCCGTGGCA |

The introduced restriction sites are underlined; the site-directed mutation sites are shown in bold italics.
